# Transmission Dynamics of Low Pathogenicity Avian Influenza Infections in Turkey Flocks

**DOI:** 10.1371/journal.pone.0026935

**Published:** 2011-10-26

**Authors:** Arianna Comin, Don Klinkenberg, Stefano Marangon, Anna Toffan, Arjan Stegeman

**Affiliations:** 1 Istituto Zooprofilattico Sperimentale delle Venezie, Legnaro (PD), Italy; 2 Department of Farm Animal Health, Faculty of Veterinary Medicine, Utrecht University, Utrecht, The Netherlands; Centers for Disease Control and Prevention, United States of America

## Abstract

Low pathogenicity avian influenza (LPAI) viruses of H5 and H7 subtypes have the potential to mutate into highly pathogenic strains (HPAI), which can threaten human health and cause huge economic losses. The current knowledge on the mechanisms of mutation from LPAI to HPAI is insufficient for predicting which H5 or H7 strains will mutate into an HPAI strain, and since the molecular changes necessary for the change in virulence seemingly occur at random, the probability of mutation depends on the number of virus replicates, which is associated with the number of birds that acquire infection. We estimated the transmission dynamics of LPAI viruses in turkeys using serosurveillance data from past epidemics in Italy. We fitted the proportions of birds infected in 36 flocks into a hierarchical model to estimate the basic reproduction number (*R*_0_) and possible variations in *R*_0_ among flocks caused by differences among farms. We also estimated the distributions of the latent and infectious periods, using experimental infection data with outbreak strains. These were then combined with the *R_0_* to simulate LPAI outbreaks and characterise the resulting dynamics. The estimated mean within-flock *R_0_* in the population of infected flocks was 5.5, indicating that an infectious bird would infect an average of more than five susceptible birds. The results also indicate that the presence of seropositive birds does not necessarily mean that the virus has already been cleared and the flock is no longer infective, so that seropositive flocks may still constitute a risk of infection for other flocks. In light of these results, the enforcement of appropriate restrictions, the culling of seropositive flocks, or pre-emptive slaughtering may be useful. The model and parameter estimates presented in this paper provide the first complete picture of LPAI dynamics in turkey flocks and could be used for designing a suitable surveillance program.

## Introduction

Infection with low pathogenicity avian influenza (LPAI) viruses is widespread and in many countries has led to outbreaks in domestic birds [1]. Although LPAI strains do not pose a serious concern for animal health, LPAI subtypes H5 and H7 may mutate into highly pathogenic strains (HPAI) [2], outbreaks of which can threaten human health [3], in addition to causing huge economic losses due to high bird-mortality rates and to the cost of control measures [4].

Although influenza viruses have been extensively studied, the current knowledge on the mechanisms of mutation from LPAI to HPAI is insufficient for predicting which H5 or H7 strains will mutate into an HPAI strain. Moreover, given that the molecular changes necessary for the change in virulence seem to occur at random [5], the probability that an LPAI strain will mutate into an HPAI strain depends on the extent of viral replication, which in turn is associated with the number of birds that acquire infection. Hence knowledge of the disease dynamics of LPAI viruses is important for better understanding their reversion to virulence. This knowledge can also contribute to optimizing surveillance systems and improving the effectiveness of control measures for reducing transmission and thus the number of virus replicates, reducing the probability of mutation into HPAI viruses.

Studies conducted on the disease dynamics of LPAI viruses under experimental conditions have provided rough estimates of the parameters of bird-to-bird transmission for a H5N2 LPAI [6] and a H7N1 LPAI [7] virus strains. For instance, the basic reproduction number (*R*_0_), which is defined as the mean number of secondary cases per primary case in a susceptible population [8] and is a key epidemiological parameter, was estimated to be between 0.6 and 4.0. However, in experimental conditions it is impossible to assess the variability in transmission that occurs among flocks in field conditions. Using outbreak data, the transmission dynamics of HPAI strains have been studied by applying compartmental models and using mortality data to extrapolate the moment of virus introduction [9], [10]. However, for LPAI epidemics, such data cannot be used because infections result in only mild symptoms and low mortality rates.

In the period 2000–2005, Italy experienced four epidemics of LPAI, all of which occurred in the north and most of which involved meat turkeys. In the present study, we used serosurveillance data from these epidemics [11] to estimate the *R_0_* of LPAI in turkeys; this is the first time that field data have been used to evaluate the transmission dynamics of LPAI. We fitted the proportions of birds ultimately infected in 36 flocks into a hierarchical model to estimate *R*_0_ and the possible variation in *R*_0_ among flocks caused by differences among farms. To obtain a more complete picture of LPAI transmission, we used experimental infection data with outbreak strains to estimate the distributions of the latent and infectious periods. These were then combined with the *R_0_* to simulate LPAI outbreaks, characterise the resulting dynamics, and discuss the implications for surveillance.

## Results

### Basic reproduction number (R_0_)

Using data from the 2000–2005 LPAI outbreaks in northern Italy, we estimated the *R*_0_ based on the seroprevalence in selected flocks after the outbreaks had come to an end (referred to as the “final size”). In other words, we considered only those flocks that tested negative to antigen detection ±5 days from the earliest positive serological finding in the flock. The selected farms consisted of those with unvaccinated meat-turkey flocks housed in a single shed. This resulted in 36 selected flocks (Table 1): 27 were infected by H7N3 and 9 by H7N1 LPAI strains. The mean seroprevalence (i.e., final size) in the selected flocks was 89.3% (Exact Fisher's 95% confidence interval: 85.7–92.2), which was significantly higher than the seroprevalence in the flocks that were positive for antigen detection (i.e., 61.7%; 95%CI: 50.3–72.3, data not shown), confirming the validity of this inclusion criterion (i.e., negative for antigen detection).

**Table 1:** Outbreak data included in the analyses.

outbreak ID	virus strain	sampled birds	positive findings
1	H7N3	10	10
2	H7N3	10	10
3	H7N3	10	5
4	H7N3	10	10
5	H7N3	10	9
6	H7N3	10	10
7	H7N3	10	9
8	H7N3	10	9
9	H7N3	10	10
10	H7N3	10	9
11	H7N1	10	10
12	H7N3	10	10
13	H7N3	10	9
14	H7N3	10	10
15	H7N3	8	8
16	H7N3	10	10
17	H7N3	10	10
18	H7N1	20	20
19	H7N1	10	8
20	H7N1	10	9
21	H7N1	10	10
22	H7N3	10	10
23	H7N1	15	12
24	H7N3	10	10
25	H7N1	10	1
26	H7N3	10	10
27	H7N1	10	10
28	H7N1	10	8
29	H7N3	10	10
30	H7N3	10	9
31	H7N3	10	7
32	H7N3	10	10
33	H7N3	10	10
34	H7N3	10	10
35	H7N3	10	2
36	H7N3	10	9

**Figure 1:**
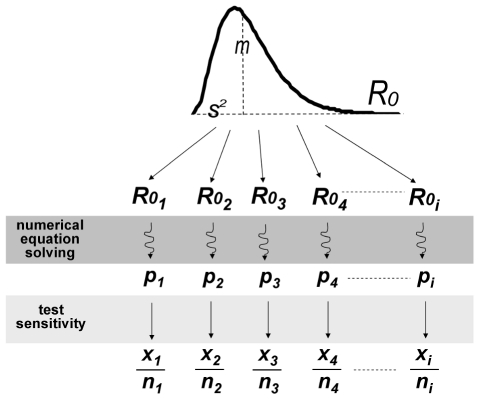
Hierarchical model linking serosurveillance data with *R_0_* in the population of infected flocks, through the final size equation. ***m***, mean *R_0_* in the population of infected flocks; ***s^2^***, variance of *R_0_* in the population of infected flocks; ***R_0i_***, basic reproductive number of each infected flock *i*; ***p***, final size of the epidemic; *x*, proportion of positive samples; ***n***, total number of samples.

The final size data were fitted into a Bayesian hierarchical model (Figure 1) to estimate the distribution of *R_0_* among flocks, resulting in a mean value of 5.5 (95% posterior credible interval: 3.4–18.3) and a variance of 11.3 (95%PCI: 1.7–298). The sensitivity of the diagnostic test (i.e., haemoagglutination inhibition) was estimated in the same model and was equal to 0.977 (95%PCI: 0.953–0.992) (Table 2), which was insensitive to the choice of prior distribution (0.975 with uninformative prior).

**Table 2:** Estimation of *R_0_*.

	median	95% posterior credibility interval
*ρ*, rate	2.73	[0.9839 – 7.47]
*κ*, shape	0.4909	[0.06023 – 2.07]
*m*, mean *R_0_*	5.535	[3.357 –18.33]
*s^2^*, variance of *R_0_*	11.29	[1.684 – 298.8]
*Se*, test sensitivity	0.9768	[0.9532 – 0.9924]

Median and 95% credibility intervals of the posterior densities of *shape* and *rate* (i.e., the parameters defining the gamma distribution of *R_0_* in the population of infected flocks), mean and variance of *R_0_* and test sensitivity.

### Estimation of latent and infectious periods

Given that field data were not available for estimating the duration of the latent and infectious periods, we used previous data from experimental infections with outbreak strains. The data were available for 18 unvaccinated commercial turkeys challenged with two different LPAI strains (H5N2 and H7N3, 9 birds per strain) and swabbed at days 3, 5, 7, 10, 12, 15 and 20 post inoculation. Infectivity was tested by means of both PCR and virus isolation assays. The test results are given in Table 3. Because sensitivity was higher for PCR, compared to virus isolation assays, we used the PCR results for our default analysis. However, given that positive virus isolation may better reflect infectivity, we repeated the analysis with the virus isolation results to assess the sensitivity of this choice for the outbreak simulations described below.

**Table 3:** Test results of swabbed turkeys at different days post infection.

		results of PCR assay (dataset A)								results of virus isolation (dataset B)						
	days p.i.*	3	5	7	10	12	15	20	days p.i.	3	5	7	10	12	15	20
ID of challenged birds	k1	+	+	+	–	–	–	–	k1	+	–	–	–	–	–	–
	k2	–	+	+	+	–	–	–	k2	–	–	+	–	–	–	–
	k3	–	+	+	–	–	–	–	k3	–	–	–	–	–	–	–
	k4	–	+	+	+	–	–	–	k4	–	–	+	–	–	–	–
	k5	–	+	+	–	–	–	–	k5	–	–	–	–	–	–	–
	k6	+	+	+	–	–	–	–	k6	–	–	–	–	–	–	–
	k7	–	+	+	–	–	–	–	k7	–	–	+	–	–	–	–
	k8	–	+	+	–	–	–	–	k8	–	+	–	–	–	–	–
	k9	–	+	+	+	–	+	–	k9	–	+	+	–	–	–	–
	k10	–	+	+	+	–	+	–	k10	–	–	–	–	–	–	–
	k11	+	+	+	+	+	–	–	k11	–	–	–	–	–	–	–
	k12	+	+	+	+	–	–	–	k12	–	–	–	–	–	–	–
	k13	+	+	+	+	+	+	–	k13	–	–	–	–	+	+	–
	k14	+	+	+	+	–	+	–	k14	–	–	–	–	–	+	–
	k15	+	–	–	–	–	–	–	k15	+	–	–	–	–	–	–
	k16	+	+	+	+	+	+	–	k16	+	–	–	–	–	+	–
	k17	+	+	+	+	–	–	–	k17	–	–	–	–	–	–	–
	k18	+	+	+	+	+	–	–	k18	–	–	–	–	+	–	–

Birds k1 to k9 were challenged with H5N2 LPAI virus and birds k10 to k18 with H7N3 LPAI virus.

*Days p.i.  =  days post inoculation.

The estimates of latent and infectious periods were calculated using a Bayesian model, and the results varied according to the definition of “infected animal”. When the definition was based on the quantity of viral genome in faeces (identified by PCR), the mean latent period was 2.9 days (95%PCI: 2.4–3.4), and the mean infectious period was 8.2 days (95%PCI: 6.5–10.6). When the definition was based on the isolation from faeces of a live virus capable of replication (detected by virus isolation), the mean latent period was 8.7 days (95%PCI: 3.9–33.8) and the mean infectious period was 2.3 days (95%PCI: 1.3–3.5) (Table 4).

**Table 4:** Estimates of the latent (LP) and infectious (IP) periods.

	dataset A		dataset B	
mean latent period (days)	2.932	[2.407; 3.388]	8.650	[3.847; 33.780]
mean infectious period (days)	8.161	[6.454; 10.580]	2.323	[1.303; 3.530]
*κ_L_*	17.480	[3.011; 128.20]	0.878	[0.240; 3.458]
*ρ_L_*	5.954	[1.096; 43.110]	0.102	[0.011; 0.533]
*κ_I_*	4.640	[2.036; 9.634]	3.803	[0.672; 53.210]
*ρ_I_*	0.568	[0.233; 1.228]	1.723	[0.332; 18.100

Median and 95% credibility intervals of the posterior densities of *κ_L_, ρ_L,_ κ_I_* and *ρ_I_* (i.e., the parameters defining the gamma distribution of LP and IP), and the mean latent and infectious periods.

### Outbreak simulations

To characterize the dynamics of LPAI outbreaks, we simulated 1,000 outbreaks in flocks of 10,000 turkeys each, with a SEIR stochastic model using the posterior median transmission parameters (Tables 2, 4, 5), with the estimates of latent and infectious periods derived from the PCR results (dataset A).

**Table 5:** Input parameters and assumptions for the three simulation models.

	model 1	model 2*	model 3
input parameters	*m*, *κ_L_*, *ρ_L_, κ_I_*, *ρ_I_*	*κ*, *ρ*, *κ_L_*, *ρ_L_, κ_I_*, *ρ_I_*	*κ*_[i]_, *ρ*_[i]_, *κ_L_*_[i]_, *ρ_L_*_[i]_, *κ_I_*_[i]_, *ρ_I_*_[i]_
basic reproduction number	*R_0_*_[i]_ = *m*	*R_0_*_[i]_ ∼ Gamma(*κ*, *ρ*)	*R_0_*_[i]_ ∼ Gamma(*κ*_[i]_, *ρ*_[i]_)
latent period	*LP*_[i]_ ∼ Gamma(*κ_L_*, *ρ_L_*)	*LP*_[i]_ ∼ Gamma(*κ_L_*, *ρ_L_*)	*LP*_[i]_ ∼ Gamma(*κ_L_*_[i]_, *ρ_L_*_[i]_)
infectious period	*IP*_[i]_ ∼ Gamma(*κ_I_*, *ρ_I_*)	*IP*_[i]_ ∼ Gamma(*κ_I_*, *ρ_I_*)	*IP*_[i]_ ∼ Gamma(*κ_I_*_[i]_, *ρ_I_*_[i]_)

*baseline model.

***m***, mean *R_0_*; ***κ_L_*** and ***ρ_L_***, parameters describing the gamma distribution of the latent period; ***κ_I_*** and ***ρ_I_***, parameters describing the gamma distribution of the infectious period; ***κ***, *shape* parameter of the gamma distribution of *R_0_*; ***ρ***, *rate* parameter of the gamma distribution of *R_0_*; i  =  1 to 1,000 (i.e., number of simulated outbreaks).

The descriptive statistics of the simulated outbreaks using the baseline model (model 2) are shown in Table 6. The quoted intervals are the 2.5^th^ and 97.5^th^ percentiles. The duration of outbreaks (i.e., from the first to the last infected turkey) ranged from 56 to 337 days (i.e., 2 to 11 months), although 90% of the infections were observed in a period of 10–150 days. The epidemic peak (i.e., the day that the peak number of infective birds was reached) occurred at a median of 45 days after the first case, which is only 7 days after a serological sample of 10 turkeys would be detected with 50% probability (median *T_det50%_* is 38 days). At the peak, a median of about 50% of the turkeys were infected, though this percentage greatly varied among farms (3% – 74%). At that time, 15.8% – 28.3% of the turkeys were already seropositive, indicating a period of overlap where both antigen and serological tests were able to detect infection. As expected, the seroprevalence at the end of the outbreak (*R_final_*,) (i.e., the final size) was high, even higher than 99.4% in half of the cases.

**Table 6:** Descriptive statistics for 1,000 simulated outbreaks using the baseline model (model 2) and reference dataset (dataset A) (i.e., PCR results).

*parameter*	mean	median	2.5^th^ percentile	97.9^th^ percentile
*Duration* (days)	106	83	56	337
*T_peak_* (days)	57	45	28	164
*D*_9*0%*_ (days)	32	20	10	150
*I_peak_* (%)	46.4	49.8	3.4	74.1
*R_peak_* (%)	28.8	29.6	15.8	28.3
*R_final_* (%)	93.5	99.4	41.9	100
*T_det50%_* (days)	47	38	25	130

Model 2 assumed that all 1,000 simulated outbreaks had the same value of mean latent and infections periods (estimated using the results of PCR assay), whereas values of *R*_0_ were all sampled from the same gamma distribution with parameters *κ* and *ρ* at the median value of the posterior distributions. Parameters' meaning: ***Duration***, duration of the epidemic in days; ***T_peak_***, time of the epidemic peak (days after infection); ***D*** **_9_**_***0%***_, time interval (days) during which the mid-90% of the cases occur (90% incidence interval); ***I_peak_***, peak number of infective birds; ***R_peak_***, seroprevalence at the epidemic peak; ***R_final_***, seroprevalence at the end of the outbreak; ***T_det50_***, time by which a serological sample of 10 turkeys would result in detection with 50% probability (days).

### Sensitivity analysis

**Figure 2:**
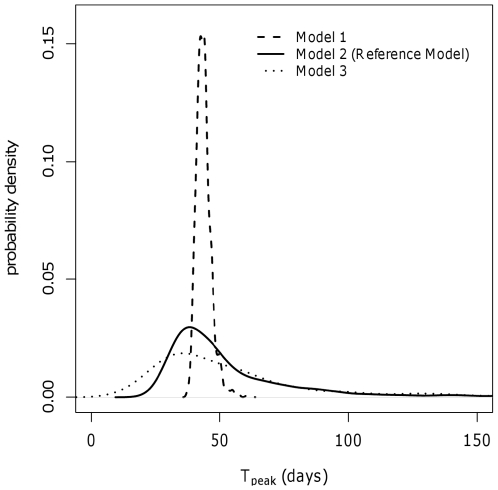
Sensitivity analysis: estimates of time of the epidemic peak (T_peak_) resulting from 1,000 outbreak simulations using the three different models and the reference dataset (i.e., PCR data).

To investigate the possible sources of variation in the outbreaks' descriptive statistics, we compared the above-mentioned results (obtained with the baseline model) with simulations derived from models with different levels of uncertainty (Table 5). In particular, model 1 was used to investigate only stochastic effects, model 2 (i.e., the baseline model) to investigate stochastic effects and variation in *R*_0_ among flocks, and model 3 to investigate stochastic effects, variation in *R_0_* among flocks, and uncertainty about the parameters that defined the distribution of the *R_0_*, latent and infectious periods. The results are provided in Table 7. The estimated median values obtained with model 1 were very similar to those obtained with the baseline model, though with a marked narrowing of the 95% credible intervals. This is clearly visible for the peak number of infective birds (median *I_peak_*: 52.4% in model 1 versus 49.8% in model 2), for which the precision of the estimation in model 1 reached a very narrow interval (51.3%–53.4%). This implies that most variation in the field is due to intrinsic differences among flocks and not to stochastic effects. Obviously, model 3 added more uncertainty to the estimates, resulting in broader credible intervals; however the median results of model 3 were similar to those obtained with model 2 (Tables 6 and 7). The differences between models 2 and 3 were relatively small, indicating that more precise parameter estimates would improve the predicted dynamics of LPAI outbreaks only to a limited extent. This can also be seen in Figure 2, in which an example of the impact of the three models on the time of the epidemic peak is illustrated. Whereas the median estimates were quite similar, the higher precision of model 1 led to a sharper distribution compared to the distributions resulting from models 2 and 3, which encompassed more uncertainty.

**Table 7:** Sensitivity analysis: descriptive statistics for 1,000 simulated outbreaks under different model assumptions and datasets.

	Model 1 – dataset A			Model 3 – dataset A			Model 2 – dataset B		
*Parameter*	median	2.5^th^ percentile	97.5^th^ percentile	median	2.5^th^ percentile	97.5^th^ percentile	median	2.5^th^ percentile	97.5^th^ percentile
*Duration* (days)	79	72	90	86	42	400	135	100	400
*T_peak_* (days)	43	39	51	47	17	235	41	18	175
*I_peak_* (%)	52.4	51.3	53.4	46.7	1.4	87.2	11.3	1.2	16.2
*R_final_* (%)	99.6	99.5	99.7	99.3	38.4	100	99.5	49.7	100

**Model 1** assumed that all 1,000 simulated outbreaks had the same *R*_0_, *κ_L_, ρ_L_, κ_I_* and *ρ_I_*, all medians from the posterior distributions. **Model 2** assumed all simulations with the same *κ_L_, ρ_L_, κ_I_* and *ρ_I_*, but with different *R*_0_. In **model 3** all simulations had different *κ_L_*, *ρ_L_*, *κ_I_*, *ρ_I_*, and *R*_0_. **Dataset A** includes the results of PCR assay, whereas **dataset B** includes the results of virus isolation. ***Duration***, duration of the epidemic in days; ***T_peak_***, time of the epidemic peak (days after infection); ***I_peak_***, peak number of infective birds; ***R_final_***, seroprevalence at the end of the outbreak.

**Figure 3:**
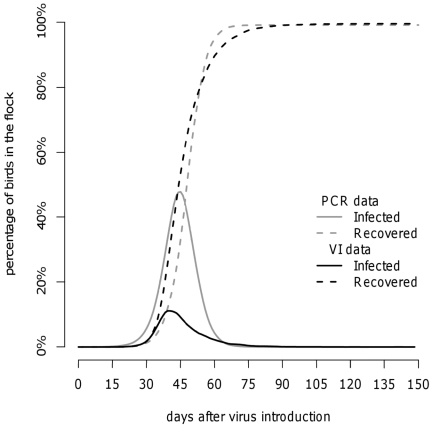
Outbreak simulation in a flock of 10,000 turkeys using the baseline model (model 2): comparison of disease dynamics assuming different definitions of “infective birds” [i.e., based on PCR data or virus isolation (VI) data].

To investigate the effect of the definition of “infectious bird” on the disease dynamics, we performed additional simulations using the posterior estimates derived from virus isolation results (i.e., dataset B) (Table 4). Table 7 shows the descriptive statistics of the 1,000 simulated outbreaks using model 2 and dataset B. The final size of the epidemic was the same (99.5%), yet the duration of the epidemic was longer (135 days), the epidemic peak occurred slightly earlier (41 days after infection), and the proportion of infectious birds at the epidemic peak was lower (11.3%) The different disease dynamics associated with different definitions of “infective birds” is shown in Figure 3. The different assumptions of infectivity (i.e., based upon different diagnostic assays) led to different peak prevalences; however, the timing of the peak prevalences was very close (41 versus 45 days after infection).

## Discussion

In this study, we provide quantitative information on key epidemiological parameters of LPAI dynamics in turkeys, which is the first time that this has been done using outbreak data. We first estimated the basic reproduction number of LPAI infections using the final size equation. To do so, some conditions had to be met. First, the data had to refer to a single population with homogeneous mixing. Because data from farms with multiple sheds were not stratified by shed, only the flocks consisting of a single shed were included in the analyses. Second, the seroprevalence in the samples needed to be representative of the entire flock, so that the seroprevalence in each sample could be considered to have a binomial distribution depending on the final size and the sample size. In accordance with the surveillance plan, sampled animals were randomly selected within each flock. Third, the outbreaks in the flocks had to have ended (i.e., no virus should have still been circulating). For this reason we included only flocks with negative virus tests ±5 days from the day of serological positivity. The validity of this inclusion criterion was indicated by the lower seroprevalence in virus-positive flocks, although absolute certainty about the final size status of the flocks can never be obtained. If a virus had still been circulating in some flocks, the *R*_0_ would have been underestimated.

Based on these assumptions, the estimated mean within-flock *R_0_* in the population of infected flocks was 5.5, meaning that on average an infectious bird would infect more than five susceptible animals. In an experimental study of van der Goot et al. (2003) [6], estimates of *R_0_* for LPAI H5N2 in chickens were much lower, ranging from 0.6 to 1.2. On the other hand, Gonzales et al. (2011) [7] recently estimated *R_0_* for LPAI H7N1 in experimentally infected chickens to be about 4.0, demonstrating a high variability in virus transmission among different strains. Another possible explanation for the difference between our results and the estimates reported in literature could be due to differences in susceptibility between chickens and turkeys, which has been reported in comparative experimental studies [12], [13] that show that turkeys are highly susceptible to LPAI infections and that chickens are less susceptible. Lastly, the difference could also be due to differences between experimental and field conditions, as reported by Bos et al. (2010) [14] for HPAI; in particular, whereas experiments take place under controlled settings, in field conditions other factors can enhance transmission, such as concurrent infections, climatic and environmental factors, and factors related to management.

In several studies on within-flock transmission of HPAI based on outbreak data [9], [10], [14], only a single *R*_0_ was estimated, based on the assumption that there is only one *R*_0_ that is common to all flocks. However, in the field a number of factors can result in differences among flocks. First of all, there are differences between LPAI virus strains [6], [7], such as the amount of virus excreted by infected birds and the minimum infectious dose [15]. Furthermore, we should also consider the differences in the characteristics of the farms and the age of the birds when the outbreak occurs. For example, the density of birds, which is mainly related to the birds' size and thus their age, can affect the contact rate among animals. Moreover, the time at which the virus enters a flock may instead influence the infectivity and/or susceptibility of the birds, which is related to their age, immunological competence and eventual stress due to intensive production cycles. Our approach took into account this variability by modelling *R_0_* as a probability distribution and thus allowing the transmission dynamics to vary from flock to flock. Furthermore, in our model, the sensitivity of the test was estimated together with *R_0_*, and the median sensitivity was 97.6%, which is fairly close to the sensitivity suggested by laboratory experience (98%). Uncertainty about test performance allowed us to better account for the fact that data came from a serosurveillance program, whose results depend on the true infectious status of the flock, the sampling scheme and the accuracy of the diagnostic assays.

To investigate the within-flock dynamics of LPAI viruses, we needed to know the temporal window of infectivity, defined by the mean lengths of the latent and infectious periods. Because this information was unavailable from field data, we based our estimates on earlier experimental infections with the outbreak strains. The infection status of single birds was tested by means of both PCR and virus isolation assays. If PCR results reflect infectivity (default), the infectious period of 8.2 days would be longer than that reported for chickens: 4.5 to 7.7 days for LPAI [6], [7] and 1.3–2.5 days for HPAI [16]. This may be related to the higher *R*_0_ in turkeys and to the virus strain.

In our experimental data, a positive PCR result indicated the presence of viral genome in faeces, which may not be sufficient for replication and the infection of new hosts, possibly resulting in an overestimate of the length of the infectious period. For this reason, we also estimated the infectious period using the results of virus isolation assay, to assess the effect on the predicted outbreak dynamics. In fact, a positive virus isolation implies that the virus can replicate and may thus better reflect infectivity. The difference between the two tests can be seen in Table 3: for example, bird k13 tested positive to PCR from day 3 to day 15 (dataset A) but showed a detectable amount of virus only starting from day 12 (dataset B). Thus, based on virus isolation, the latent period would be longer and the infectious period shorter. However, the difference in terms of mean generation time was small: 7.9 and 10.1 days for PCR and virus isolation results, respectively. The comparison of the prevalence between the two datasets is unfair because it is based on different diagnostic tests and assumptions regarding infectivity. The important difference lies in the timing of the peak prevalence and the increase in seroprevalence, which were rather similar when comparing PCR and virus isolation data (Figure 3), indicating that the results are not very sensitive to the choice of diagnostic assay.

The comparison of the three simulation models showed that the variation in *R_0_* among flocks plays an important role in the variation among outbreaks (Tables 6 and 7). In Figure 2, it appears that model 1, which only accounts for differences due to the stochastic process, resulted in only a limited variability in the timing of the epidemic peak (median: 43 days, 95%PCI: 39–51). Adding uncertainty related to possible variation of *R_0_* among flocks (model 2) resulted in a similar median estimate (45 days), yet it remarkably increased the variation (95%PCI: 28–164), as demonstrated by the much flatter density distribution of the parameter. The inclusion of further uncertainty about the parameter estimates (model 3) led to an additional widening of the interval (95%PCI: 17–235), but the difference was limited extent when compared to model 2. (Figure 2, solid versus dotted line). We could thus argue that our estimates of latency, infectivity and the mean and variance of *R_0_* in the population of infected flocks are sufficiently precise, though we cannot overlook the variation in *R_0_* among flocks, which seems to play the most important role in the variation among infected premises.

The simulations showed that the finding of seropositive birds does not necessarily mean that the flock is no longer infective: Table 6 shows that at the epidemic peak about 50% of the turkeys were infected, yet that 16% to 28% of the turkeys were already seropositive, indicating a period of overlap where both antigen and antibodies are detectable (Figure 3, grey lines). This implies that seropositive flocks may still pose a risk for other flocks; thus the enforcement of appropriate restrictions, the culling of seropositive flocks or pre-emptive slaughtering may be useful in preventing farm-to-farm transmission. On the other hand, sero-sampling for early disease detection may be difficult, because the time by which a serological sample of 10 turkeys would result in detection with 50% probability (*T_det50%_*) is only 7 days before peak infectivity (*T_peak_*).

The model and parameter estimates presented in this paper provide the first complete picture of LPAI dynamics in turkey flocks and could as such be used for the design and optimization of a suitable surveillance program.

## Materials and Methods

The within-flock disease dynamics of LPAI were investigated using field data from outbreaks and data from experimental infections and combining these data in a stochastic simulation model. The investigation was conducted in three steps:

1. Estimation of within-flock *R_0_* for LPAI infections using field data and a Bayesian hierarchical model based on the final size equation, provided below;
2. Estimation of the distribution of latent and infectious periods of LPAI in turkeys, using pre-existing data from experimental infections; and
3. Simulation of outbreaks using the estimates in points 1 and 2 and characterisation of LPAI outbreaks and their uncertainty (sensitivity analysis).

### Estimation of the basic reproduction number (R_0_)

#### Data source

The field data were provided by the intensive surveillance system which was in place during the LPAI epidemics in 2000–2001, 2002–2003, 2004 and 2005 [17]. During and around the time of the epidemics, a total of 6,102 poultry farms were routinely visited; 495 infected premises (i.e., outbreaks) were identified; 429 (87%) of these premises reared meat turkeys. Of the 429 outbreaks, we included only those that had occurred among unvaccinated flocks (n = 204). Although it would have been interesting to have investigated the disease dynamics in vaccinated birds, this was not possible because in the vaccinated flocks only unvaccinated sentinels were sampled. To fulfil the assumption of homogeneous mixing of the animals required for the analysis, we only included those farms on which the birds were housed in a single shed (n = 64).

#### Inclusion criteria

At the 64 farms, multiple samplings had been carried out. In each flock, a median of 10 (range: 8–20) birds per sampling were considered. We considered the earliest sampling that revealed a positive serological finding and determined whether an antigen detection assay had been performed ±5 days from this finding; antigen detection had been performed on mixed samples (pools) of five birds. If the flock was negative, then the outbreak was assumed to be over, and the seroprevalence in the sample was considered to represent the proportion of the population that had been infected by the end of the outbreak (defined as the “final size”). This resulted in the identification of 36 outbreaks (Table 1 and Table S1).

#### Model building

The *final size* of an epidemic (*p*, the proportion of a population that had been infected by the end of an outbreak) and the basic reproduction number (*R_0_*) are related through the final size equation:
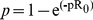
(1)which is considered to be valid under very general circumstances [18]. Serosurveillance data were fitted to a hierarchical model (Figure 1), assuming that *R_0_* in the population of infected flocks followed a gamma probability distribution, with mean *m* and variance *s^2^*. Each *R_0i_* of flock *i* corresponds to a final size *p_i_*, calculated numerically from Eq. 1. The observed number of positive samples *x_i_* in each flock was then considered to be a sample from a binomial distribution with *n*  =  *n_i_* (sample size) and *p*  =  *p_i_* (final size)*test sensitivity (which represents the apparent prevalence in each flock *i*). The gamma distribution of *R_0_* was defined by the parameters *shape* (*κ*>0) and *rate* (*ρ*>0), which are related to the mean (*m*) and variance (*s^2^*) of *R_0_* as:


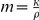
 and 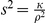


Furthermore, the use of an imperfect diagnostic test was assumed in the detection of seroprevalence, with sensitivity modelled as a Beta distribution. This model is the result of a careful preliminary investigation in which several alternatives have been compared. The initial assumption of a single R_0_ value common to all the infected flocks did not fit our field data and we thus modelled R_0_ as a probability density distribution. Different hypothesis on R_0_ distribution and test sensitivity were then explored and evaluated by means of the deviance information criterion (DIC) [19]. The currently presented model is the one which resulted in the best fit of the field data.

The model was implemented in WinBUGS software version 1.4.3; posterior distributions were obtained using the default internal Gibbs sampler [20]. Uninformative prior distributions were used for the parameters *κ* and *ρ* [i.e., Gamma(0.01,0.01)]. Informative prior information, based on laboratory experience (but no solid data), was used for the distribution of test sensitivity. Using the R function beta.prior (available at http://skoval.bol.ucla.edu/beta.prior.R), we derived the parameters of the Beta distribution that corresponded to a most likely sensitivity of 0.98 and to a 95% certainty that the sensitivity would be greater than 0.95 [i.e. Beta(151.77,4.08)]. Posterior inferences were based on 30,000 iterations with a sampling lag of 10, after a burn-in of 15,000 iterations was discarded. Convergence was assessed by running multiple chains from dispersed starting values and using the Gelman-Rubin statistic.

### Estimation of latent and infectious periods

#### Data source

The data used for this analysis were taken from a vaccine trial performed in 2004 at the Italian National Reference Laboratory for Avian Influenza (unpublished data). Eighteen unvaccinated commercial turkeys (i.e., the controls of the trial) were challenged with two LPAI strains at 12 weeks of age via the intranasal route. Nine birds were challenged with H5N2 LPAI virus A/TK/IT/80 and 9 birds with H7N3 LPAI virus A/TK/IT/8000/02. The infective dose was 10^4^ EID_50_. For each bird, cloacal swabs were taken at day 3, 5, 7, 10, 12, 15 and 20 post-inoculation and tested using a real-time RT-PCR assay and virus isolation in SPF fertile eggs. The results are given in Table 3. Because of the higher sensitivity, we used the PCR results for our default analysis. However, given that a positive virus isolation may better reflect infectivity, we repeated the analysis with the virus isolation results to assess the sensitivity of this choice for the simulation output. Thus two different datasets were built: dataset A (PCR assay) and dataset B (virus isolation).

#### Model building

We assumed that infectivity was indicated by a positive test result and that, based on individual test results (Table 3), the infectious period was preceded by a latent period. This latent period began immediately after virus inoculation (day 0) and ended at a time point (T_1_) between the last negative and the first positive test result. Consequently, the infectious period started just after the latent period and ended in the period (T_2_) between the last positive test and the subsequent negative test. For example, bird k2 in Table 3 (dataset A) showed a latent period starting at day 0 and lasting to somewhere between day 3 and 5 (3<T_1_<5); the infectious period lasted from T_1_ to between day 10 and 12 (10< T_2_<12). We then assumed that both the latent period (*LP*) and infectious period (*IP*) in the population of infected birds followed a gamma distribution, characterized by parameters *κ* and *ρ,* as follows:

*LP* ∼ gamma(*κ_L_ , ρ_L_*) and *IP* ∼ gamma(*κ_I_* , *ρ_I_*)

To have an estimate of the latent and infectious periods in the population, we built a Bayesian model to link the distributions of these periods in the population of infected birds with the test results of the 18 challenged turkeys. We noted that for each bird (k1–k18) and at each sampling day (*D* = 3, 5, 7, 10, 12, 15 and 20) the test result *y* could be either positive (1) or negative (0). It follows that *y* has a Bernoulli distribution, depending on the success probability *π*:

*y* ∼ Bernoulli(*π*)

Assuming that the diagnostic test perfectly reflects infectivity, the success probability (i.e., positive test result) depends on whether or not the sample was taken during the infectious period (i.e., when the bird sheds the virus with faeces). We thus assumed 100% probability of a positive test result (*π* = 1) if the sampling day *D* was within the infectious period and 0% probability of a positive test result (*π* = 0) if the sample was collected before or after the infectious period:

*π* = 1 if T_1_≤*D*≤T_2_ and *π* = 0 if *D* < T_1_ or *D* > T_2_

Due to the limited amount of data, it was impossible to obtain reliable estimates of LP and IP for H5N2 and H7N7 strains separately. However, preliminary investigations showed that the overall generation time was a good average of the two separately, which were not that far apart indeed. We thus decided to estimate LP and IP using all the available data, given that the further infection model will encompass enough variability to allow for different virus transmission characteristics.

The model was implemented in WinBUGS software using the default internal Gibbs sampler [20]. Uninformative prior distributions were used for the parameters *κ_L_, ρ_L,_ κ_I_* and *ρ_I_* [i.e., Gamma(0.01,0.01)]. Posterior inferences were based on 30,000 iterations with a sampling lag of 10, after a burn-in of 15 000 iterations was discarded. Convergence was assessed by running multiple chains from dispersed starting values and using the Gelman-Rubin statistic.

### Outbreak simulations

#### Model building

Estimates of *R_0_*, *κ_L_, ρ_L_*, *κ_I_* and *ρ_I_* were combined to simulate and characterize the course of LPAI outbreaks in turkey flocks. Simulations were carried out in R statistical software [21]. Simulations started with one index case infected at time  = 0 and 9,999 susceptible birds. The end of the latent and infectious periods of the index case were sampled and stored. At each time step of 0.02 days, the number of infected birds *I* was calculated; then the number of new infections *C* was sampled from a binomial distribution (*n*  =  number of susceptible birds; *p* = 0.02 *β I*/10,000; *β*  =  transmission rate  =  *R_0_ λ_I_*); finally, the latent and infectious periods of the new cases were sampled and stored.

The simulated outbreaks were summarized by calculating six descriptive statistics: the time of the epidemic peak *T_peak_*, the peak number of infective birds *I_peak_*, the seroprevalence at the epidemic peak *R_peak_*, the seroprevalence at the end of the outbreak *R_final_*, the time interval during which the mid-90% of the cases occur (90% incidence interval) *D_90%_*, and the time by which a serological sample of 10 turkeys would result in detection with 50% probability (assuming a test sensitivity of 97.7%) *T_det50_*.

#### Sensitivity analysis

To distinguish between sources of variation and uncertainty, three sets of 1,000 simulations each were performed (Table 5):

- model 1: all simulations with the same *R*_0_, *κ_L_, ρ_L_*, *κ_I_* and *ρ_I_*, all medians from the posterior distributions. Variation among these simulations only reflects stochastic effects.
- model 2: all simulations with the same *κ_L_*, *ρ_L_*, *κ_I_* and *ρ_I_*, but with different *R*_0_. The values of *R*_0_ were all sampled from the same gamma distribution with parameters *κ* and *ρ* at the median value of the posterior distributions. Variation among these simulations reflects stochastic effects plus variation in *R_0_* among flocks.
- model 3: all simulations with different *κ_L_*, *ρ_L_*, *κ_I_*, *ρ_I_*, and *R*_0_. For each simulation, a random quartet of *κ_L_*, *ρ_L_*, *κ_I_* and *ρ_I_* was sampled from their multiivariate posterior distribution. A random couple of *κ* and *ρ* was also sampled, and *R*_0_ was sampled from the corresponding gamma distribution. Variation among these simulations reflects stochastic effects and variation in *R*_0_ among flocks, plus uncertainty about the parameter values.

Model 2 was our baseline model because it reflects the course of outbreaks and variation therein, according to our best estimate. The estimates of *κ_L_*, *ρ_L_*, *κ_I_* and *ρ_I_* included in the above-mentioned models came from dataset A (i.e., PCR results) because we selected PCR as our default analysis, given its higher sensitivity. To investigate the role of the virus detection assay as a further source of uncertainty, a fourth set of 1,000 simulations was performed applying model 2 to dataset B (i.e., virus isolation results).

### Ethics statement

This study was carried out in strict accordance with the requirements of Italian Law n. 1,1.6 of 27 January 1.992 (OJ.LR. 18 February 1.992, n.40, O.S.) and further amendments referring to Council Directive 86/609/FEC of the European Community (OJ.E.C. 18 December 1986, n. 358) on the protection of animals used for experimental and other scientific purposes. The protocol met the requirements outlined in Annex 4 to Circular n. 8 of the Italian the Ministry of Health, 22 Apr-J. 1994. All animal manipulations were performed under Tiletamine HC1-Zolazepam HC1 anesthesia and all efforts were made to minimize suffering. At the end of the experiment, the animals were euthanized by terminal anaesthesia. The handling and publication of the data generated from such experiment have been approved by the Institutional Ethics Committee of the Istituto Zooproflattico Sperimentale delle Venezie (Permit Number: CE.IZSVE.01/2011).

## Supporting Information

Table S1 **Raw data used to estimate the probability density distribution of R_0_.**
(PDF)
