# Supplementary material for: Transmission Dynamics of Low Pathogenicity Avian Influenza Infections in Turkey Flocks
Source: PLoS One. 2011 Oct 26;6(10):e26935. doi: 10.1371/journal.pone.0026935 (PMC3202598; doi:10.1371/journal.pone.0026935)
Supplement: Table S1 — Raw data used to estimate the probability density distribution of R0. (PDF) [file pone.0026935.s001.pdf]

Table S1. Raw data used to estimate the probability density distribution of R_0.

flock ID	flock size	virus strain	first sampling			second sampling			third sampling			fourth sampling		
			day	serology result (positive/tested)	virology result (pool)	day	serology result (positive/tested)	virology result (pool)	day	serology result (positive/tested)	virology result (pool)	day	serology result (positive/tested)	virology result (pool)
1	8900	H7N3	92	0/10	not done	97	not done	negative	125	**10/10**	**negative**			
2	13300	H7N3	79	0/10	not done	103	not done	negative	127	**10/10**	**negative**			
3	4300	H7N3	133	**5/10**	**negative**									
4	11000	H7N3	58	0/10	negative	105	**10/10**	**negative**	147	7/10	not done			
5	8000	H7N3	82	0/10	negative	124	0/10	positive	128	0/10	negative	135	**9/10**	**negative**
6	6400	H7N3	64	0/10	negative	92	0/10	negative	99	**10/10**	**negative**			
7	13000	H7N3	73	0/10	not done	107	**9/10**	**negative**	149	not done	negative			
8	11000	H7N3	78	0/10	negative	112	**9/10**	**negative**	154	not done	negative			
9	15000	H7N3	101	not done	positive	107	0/10	positive	**109**	**10/10**	not done	**114**	not done	**negative**
10	16800	H7N3	89	0/10	negative	126	0/10	negative	131	**9/10**	**negative**			
11	10000	H7N1	77	0/10	not done	96	**10/10**	not done	99	not done	**negative**			
12	10900	H7N3	34	0/10	not done	49	not done	negative	83	**10/10**	**negative**			
13	6116	H7N3	63	0/10	not done	106	**9/10**	**negative**						
14	5200	H7N3	66	0/10	negative	117	**10/10**	**negative**	144	not done	negative			
15	5000	H7N3	73	0/10	negative	109	**8/8**	**negative**	116	9/10	negative	132	not done	negative
16	6800	H7N3	80	0/10	not done	84	not done	negative	118	**10/10**	**negative**	151	not done	negative
17	16000	H7N3	43	0/10	negative	79	**10/10**	**negative**						
18	6580	H7N1	87	0/10	positive	102	**20/20**	**negative**	110	not done	negative			
19	12600	H7N1	81	**9/10**	**negative**	93	6/10	not done						
20	12600	H7N1	81	**8/10**	**negative**	93	6/10	not done						
21	12600	H7N1	81	**10/10**	**negative**	93	3/10	not done						
22	3600	H7N3	56	not done	negative	101	**10/10**	**negative**						
23	25600	H7N1	71	**12/15**	**negative**	78	8/20	not done						
24	7600	H7N3	55	0/10	not done	76	0/10	negative	98	**10/10**	**negative**	111	10/10	not done
25	7500	H7N1	51	0/10	not done	64	**1/10**	**negative**						
26	7800	H7N3	84	0/10	negative	102	**10/10**	**negative**						
27	4000	H7N1	81	0/10	not done	86	0/15	negative	106	0/10	not done	110	**10/10**	**negative**
28	6400	H7N1	**87**	**8/10**	not done	**91**	not done	**negative**	98	8/10	not done			
29	12000	H7N3	88	10/10	not done	92	**10/10**	**negative**	112	not done	negative	115	not done	negative
30	17760	H7N3	65	0/10	not done	131	**9/10**	**negative**	145	7/10	negative	147	not done	negative
31	17760	H7N3	65	0/10	not done	131	**7/10**	**negative**						
32	12000	H7N3	42	not done	negative	77	0/10	negative	106	**10/10**	**negative**			
33	12320	H7N3	103	0/10	not done	125	0/10	negative	137	not done	negative	152	**10/10**	**negative**
34	16000	H7N3	56	not done	negative	84	0/10	negative	112	**10/10**	**negative**			
35	9800	H7N3	71	0/10	negative	113	0/10	negative	136	**2/10**	**negative**	137	10/10	negative
36	15300	H7N3	91	0/10	not done	112	0/10	negative	140	**9/10**	**negative**			

Legend: **day** indicates the day of sampling, starting from the onset of the production cycle; the **shaded cells** corresponds to the data included in the analysis (i.e., earliest positive serological result associated to a negative virological result within ±5 days).
